# Authors’ Response to Peer Reviews of “Eye Care Service Use and Associated Health-Seeking Behaviors Among Malawian Adults: Secondary Analysis of the Malawi Fifth Integrated Household Survey 2019-2020”

**DOI:** 10.2196/57620

**Published:** 2024-04-09

**Authors:** Thokozani Mzumara, Marios Kantaris, Joseph Afonne

**Affiliations:** 1 Department of Optometry, Mzuzu University, Mzuzu, Malawi; 2 Department of Ophthalmology, Mzimba North District Hospital, Ministry of Health, Mzuzu, Malawi; 3 Unicaf University, Lusaka, Zambia; 4 Health Services and Social Policy Research Centre, Nicosia, Cyprus

**Keywords:** access to health, health service utilization, eye care use, health-seeking behavior, sociodemographic determinant, visual impairment, social support, women empowerment, education, eye care, eye, ophthalmology, visual, ECU, eye service, utilization, Malawi, empowerment, health service use, use

*This is the authors’ response to peer-review reports for “Eye Care Service Use and Associated Health-Seeking Behaviors Among Malawian Adults: Secondary Analysis of the Malawi Fifth Integrated Household Survey 2019-2020.”*

## Round 1 Review

### Anonymous [1]

*Potentially an interesting paper [* *2* *], but more details are needed in the methods to enable the reader to understand the results.*

*The English is not good throughout the document, and the writing could be much more succinct and precise.*

Response: We have reviewed the language problems.

### Anonymous [3]

#### General Comments

*This paper is a secondary analysis of a Malawian household survey exploring associations of patients who self-reported as having used formal eye care services. It is a useful idea to use this survey data for this purpose, but the author needs to check that they are using the correct source numbers for their statistical analysis and only report the numbers actually surveyed—not the national estimated numbers derived from these.*

#### Specific Comments

##### Major Comments

*1. “In Malawi, 3.3% of the population is blind compared to 1.01% in America [4,5].” There is no way 3% of Malawi is blind. (Half of Malawi’s population are children, so if 3% of Malawi was blind, that would be about 1 in 20 adults—not possible.) Check your references.*

Response: Done.

*2. The abstract needs improving to give the definition of eye care use (ECU). In the results, it says “The prevalence of ECU was 60.6%,” which is not really a prevalence unless you give a clearer definition, for example “of those with eye symptoms, what proportion have access formal eye care services in the two weeks prior to the survey date.”*

Response: Done.

*3. The sample was 28,388 adults? You cannot, then, in the results’ “Characteristics of study participants” section say there were 6 million young adults involved or that 5,660,836 (56%) of the adults were married. You also can’t say that “27,336 (0.3% of 2,734,768) complained of ocular symptoms.” This is the main problem with the report—you need to give the actual numbers of people surveyed who reported ocular symptoms—presumably 0.3% of 28,388—which is only 85 people. Thus your CIs/other statistical analyses around estimates with a sample of 85 people reporting eye symptoms will be quite different than if you extrapolate to the whole population of Malawi.*

Response: We have removed the section “characteristics of the study participants.”

##### Minor Comments

*4. “We entered the variables...”: Who is “we”? I only see one author*.

Response: We have added other authors as initially indicated on the system database.

*5. “Sort care” should be “sought care”: This is used 5 times in the paper so should be changed at all uses.*

Response: We have corrected the error.

*6. There are some random capital letters in various places: “that In Malawi”—why has “In” got a capital?*

Response: We have corrected the error.

## Round 2 Review

*Point-by-point response to decision letter.*

### Methods

*1. Please justify the use of the confusion matrix in the manuscript.*

Response: We have withdrawn use of the confusion matrix from the paper.

*2. Please add the age of people included in the Integrated Health Survey (IHS).*

*3. Was this a national survey? If not, please specify the location.*

Response: This was a national survey including all districts in the country.

*4. Results: Please add the number of households and individuals included in the analysis.*

Response: We have added the number of households and individuals included in the study

*5. The sentence starting “But it was...” is not clear. Please rewrite.*

Response: We have edited this part.

### Main Text

#### Introduction

*6. Most of the key elements are covered, but it is poorly written. The following paper might be another useful reference: Tafida A, Kyari F, Abdull MM, et al. Poverty and blindness in Nigeria: findings from the national survey of visual impairment and blindness. Ophthalmic Epidemiol. 2015;22(5):333-41 [doi: 10.3109/09286586.2015.1077259] [Medline: 26395660]*

Response: We have rewritten the introduction and included the reference suggested above.

*7. Please clarify the aims and objectives of the study. For example, results on reasons for not accessing services are presented, but this is not mentioned beforehand.*

#### Methods (Additional Feedback)

*8. To better understand the source of data used in the analysis, please explain the sample size calculation and the sampling strategy for IHS. Presumably, households are the last sampling unit; are all eligible individuals normally living in the selected households then interviewed?*

Response: We have explained in the text.

*9. The paragraph on sampling weights is not clear. Presumably, this reflects the sampling strategy for the IHS so that the findings can be extrapolated to the whole population aged ≥15 years? If so, please explain.*

Response: We have included this explanation in the text.

*10. Please outline what confusion matrix techniques are for, what the method entails, and the outputs of the analysis.*

Response: We have removed this from the article.

*11. What symptoms were used in the analysis?*

Response: We have indicated symptoms related to eye problems.

*12. “Data” is plural: should say “data were....”*

Response: We have edited.

#### Results

*13. Define young adults and older adults.*

Response: We have edited.

*14. I assume Table 1 shows findings extrapolated to the whole country. The results of the IHS should be presented first, followed by extrapolations to the whole country, specifying the weights used, so that the analysis is more transparent.*

Response: We have presented the results of IHS without the weighting before extrapolations under Multimedia Appendix 1.

*15. Table 1: Put the data into a proper table, as the results are difficult to see at the moment.*

Response: We have modified Table 1 and included a write up for the the rest.

*16. The results need to be better ordered. At the moment regional differences in prevalence are included in the paragraph on where participants sought care.*

Response: We have rearranged.

*17. I do not understand the findings in Table 3. Presumably, these are the findings from the confusion matrix technique?*

Response: We have removed Table 3.

*18. It would be interesting to know whether the nature of the symptoms reported influenced health-seeking behavior. For example, were those who reported loss of vision more likely to access services?*

Response: This was beyond the scope of this study, as the data did not cover aspects of vision loss.

#### Discussion

*19. Please put negligence in inverted commas, as this is a judgmental term that means failure to give enough care or attention to someone or something that you are responsible for.*

Response: We have modified.

*20. An important reason why chronic conditions were more common than other conditions, which are often short-lived, is because of the cross-sectional nature of the study and the short 2-week period over which participants were to report eye conditions.*

Response: We have included this suggestion in the text.

#### References


*21. Follow instructions to authors. For example, how many authors should be quoted (ref 1 in particular)?*


Response: We have corrected it.

## Abbreviations

ECU: eye care use
IHS: Integrated Health Survey
